# Memphis FitKids: implementing a mobile-friendly web-based application to enhance parents’ participation in improving child health

**DOI:** 10.1186/s12889-018-5968-6

**Published:** 2018-08-29

**Authors:** Gerhild Ullmann, Satish K. Kedia, Ramin Homayouni, Cem Akkus, Michael Schmidt, Lisa M. Klesges, Kenneth D. Ward

**Affiliations:** 1 Social and Behavioral Sciences, School of Public Health, University of Memphis, 3825 Desoto Ave, Memphis, TN 38152 USA; 2 Bioinformatics, Biological Sciences, College of Arts and Sciences, University of Memphis, Memphis, TN USA; 3 Art, College of Communication and Fine Arts, University of Memphis, Memphis, TN USA; 4 Epidemiology, School of Public Health, University of Memphis, Memphis, TN USA

**Keywords:** Parenting, Healthy lifestyle, Childhood obesity, Overweight adolescents, Website development, Geographic information system, Internet, Physical activity, Social marketing, Health promotion, Health promotion

## Abstract

**Background:**

Child obesity is a major public health challenge, increasing the risk of chronic medical conditions such as type 2 diabetes, metabolic syndrome, and hypertension. Among U.S. states, Tennessee has one of the highest rates of child obesity. Emerging communication technologies can help to deliver highly disseminable population-level interventions to improve health behavior. The aim of this paper is to report the implementation and the evaluation of the reach of Memphis FitKids, a web-based application, intended to promote healthy behaviors for families and children.

**Methods:**

A community-level demonstration project, Memphis FitKids, was developed and implemented in Tennessee’s Greater Memphis Area. This application (www.memphisfitkids.org) was designed for parents to assess their children’s obesity risk through determinants such as weight, diet, physical activity, screen time, and sleep adequacy. A built-in “FitCheck” tool used this collected information to create a report with tailored recommendations on how to make healthy changes. A Geographic Information Systems component was implemented to suggest low-cost neighborhood resources that support a healthy lifestyle. A social marketing framework was used to develop and implement FitKids, and a Community Advisory Board with representatives from community partners (e.g., the YMCA of Memphis, the Pink Palace Family of Museums, and the Memphis Public Library) supported the implementation of the project. Five kiosks distributed in the community served as public access points to provide a broad reach across socioeconomic strata. Presentations at community events and the use of Facebook facilitated the promotion of FitKids. Website traffic and Facebook usage were evaluated with Google Analytics and Facebook Insights, respectively.

**Results:**

In Tennessee, 33,505 users completed 38,429 FitCheck sessions between July 2014 and December 2016. Among these, 6763 sessions were completed at the five kiosks in the community. FitKids was presented at 112 community events and the social media posts reached 23,767 unique Facebook users.

**Conclusions:**

The Memphis FitKids demonstration project showed that web-based health tools may be a viable strategy to increase access to information about healthy weight and lifestyle options for families. Mobile-friendly web-based applications like Memphis FitKids may also serve health professionals in their efforts to support their clients in adopting healthy behaviors.

**Electronic supplementary material:**

The online version of this article (10.1186/s12889-018-5968-6) contains supplementary material, which is available to authorized users.

## Background

Child and adolescent obesity continues to pose a serious public health threat in the United States. National Health and Nutrition Examination Survey data indicated that approximately 17% of youth were obese in 2011–2014 [1, 2], including 8.9% of 2–5 year olds, 17.5% of 6–11 year olds, and 20.5% of 12–19 year olds. Despite a reduction in the obesity rate among 2–5 year olds from 13.9% in 2003–2004 to 8.9% in 2011–2012 [3], overall obesity rates among children and adolescents in the United States remained high [4]. Additionally, there are ethnic and geographic differences in obesity rates for these age groups. During 2011–2014, rates were highest among Hispanic youth (21.9%), followed by non-Hispanic black (19.5%) and non-Hispanic white (14.7%); the lowest prevalence was observed among non-Hispanic Asian youth (8.6%) [2]. Furthermore, a meta-analysis including pooled data of 74,168 youth aged 2–19 reported that those living in rural areas have 26% higher odds of obesity, compared to urban youth [5].

Tennessee is one of seven states with the highest rates of overweight and obesity among high school students. In 2013, 15.4% of Tennessee’s high school students were overweight and 16.9% were obese [6]. Obesity rates are especially high among high-schoolers from minority racial and ethnic groups, including Hispanics and African Americans. Memphis - the second-largest metropolis in Tennessee and the county seat of Shelby County - is one of the most obese counties in the state. According to the 2012–2014 Shelby County Community Health Assessment, 33.4% of the county’s adult population was obese [7]. The 2013 Youth Risk Behavior Surveillance System (YRBSS) showed that 18% of Memphis’ high-schoolers were overweight and 19.2% were obese compared to 16.6% and13.7% nationally [8].

Major behavioral risk factors for childhood obesity are well known and are largely modifiable. Most children and adolescents do not engage in the recommended 60 min per day of physical activity [9]. According to the YRBSS, nationwide only 27.1% of high school students met the physical activity recommendations in 2015 while their screen time remained high [8]. On average school days, 24.7% of the high school students watch TV and 41.7% use video games or the computer for things not related to school for more than 3 h [8]. Children are at much higher risk of being overweight or obese if they follow a sedentary lifestyle and do not participate in sports or other physical activities outside of school [6, 10]. Unhealthy diet patterns, sugary beverages, and large portion sizes contribute to childhood obesity [6, 11].

The implications of this epidemic are startling. The increase in childhood obesity is linked to a dramatic rise in the number of children suffering from type 2 diabetes. The estimated prevalence of type 2 diabetes among American children and youth increased by 30.5% between 2001 and 2009 [12]. Of the 3679 students in Tennessee’s schools who were diagnosed with diabetes, 19.7% had type 2 diabetes [13]. If current trends continue, children with type 2 diabetes may experience coronary heart disease and heart failure as young as 30 or 40 years of age, adding billions of dollars in healthcare expenditure [14]. Other chronic medical conditions such as, hypertension, metabolic syndrome, digestive diseases, gallstones, and obstructive sleep apnea among children are already on the rise [15, 16].

A multitude of intervention programs have been designed and implemented to address childhood obesity, the majority being school-based [17–21]. Despite the benefits of implementing interventions in school settings, a meta-analysis emphasized the need for interventions that use new technologies and include families and communities [22]. Emerging information and communication technologies may have the potential for delivering interventions [23, 24] in a cost-effective manner to improve health behavior [25].

Web-based technologies designed to promote healthy lifestyle changes offer a variety of supportive tools. Reviews analyzing web-based interventions that focused on behavior or lifestyle change in adults with type 2 diabetes found that the majority of interventions aimed to increase physical activity and/or to improve diet [26, 27]. Although there are not yet any evaluations of population-level web-based interventions, a recent meta-analysis examined the effectiveness of randomized controlled trials delivered by mobile devices on physical activity and sedentary behavior. The authors reported positive intervention effects for total physical activity and moderate-to-vigorous intensity physical activity, but the pooled effects did not reach statistical significance. Compared with usual care, the results indicated a statistically significant greater reduction of sedentary behavior after completing the technology supported interventions (standard mean difference − 0.26, 95% confidence interval − 0.53 to − 0.00) [28]. These internet-based interventions included features such as progress tracking, logbooks, email counseling, tools for goal setting, platforms for networking or peer support (online communities, chats, and message boards) and other general resources. Most internet-based studies are unrelated to any geographic location and hence are not able to provide users with local community resources [26].

Most internet-based applications target adults; a recent report about an on-going randomized controlled trial using a web-based application with downloadable software for smartphones is promising [29]. This study examined the effects of a 6-month parental intervention on health behaviors of 300 Swedish preschoolers. The intervention group received an app called ‘Mobile-based Intervention Intended to Stop Obesity in Preschoolers’ (MINISTOP), while the control group received written information about healthy living. The app was tested with a small sample of parents and provided information about diet and physical activity. Parents can log their child’s behavior and receive weekly feedback. In addition, parents can contact a psychologist and dietician, if they have any questions.

Overall, web-based applications seem to be more effective if they are developed based on a theoretical framework [26]. The Social Marketing (SM) framework has been widely used in public health for intervention, policy development, health promotion initiatives, and campaigns. [30–32]. Several social marketing-based interventions have been implemented to promote behavioral changes in settings such as schools, workplaces, churches, and communities [33, 34]. These health campaigns and interventions addressed a wide range of issues including consuming healthy diet, increasing physical activity, improving health literacy, prevention or cessation of tobacco, drug or alcohol use, and the promotion of health screenings in various populations [34]. Well-known for successfully using the SM framework is the VERB campaign, a multiethnic media campaign to increase physical activity among children aged 9–13 years [35–39]. Supported by a $125 million grant, VERB was able to launch a large campaign with a multilingual website, print material, community events, and TV and radio spots. The results of the VERB campaign indicated a significant increase of physical activity in children in this age group [38, 40–42]. Thus, population-level web-based interventions show promise to improve children’s health, but to date no such interventions have targeted multiple behaviors (e.g., diet, physical activity, screen time, and sleep), provided tailored feedback on risk and behavior change goals, or attempted to link parents to local resources by using Geographic Information Systems (GIS).

**Fig. 1:**
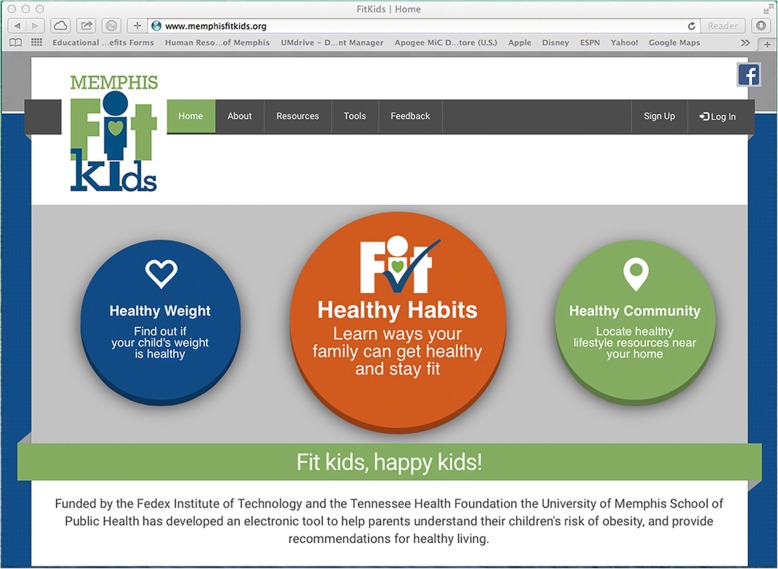
FitKids Website www.memphisfitkids.org

To address the childhood obesity epidemic in the local community, the University of Memphis’ School of Public Health used the SM framework to develop a free, innovative mobile-friendly web-based application, “Memphis FitKids.” The program was developed by a multidisciplinary team consisting of experts from public health, health promotion, health informatics, GIS, graphic design, nursing, exercise science, and nutrition. Memphis FitKids (www.memphisfitkids.org) (Fig. 1) was designed for parents to assess their children’s weight, growth pattern, diet, physical activity, screen time, and sleep adequacy through a “FitCheck” tool that was created for this project based on clinical practice guidelines and consensus panel recommendations [9, 43–46].

Based on FitCheck results, parents received personalized recommendations on how to make lifestyle changes in their family to reduce their child’s risk of obesity and to support healthy behaviors. In addition, FitKids utilized GIS technology to provide parents with information about resources in their neighborhoods to promote physical activity and healthy eating.

The purpose of the Memphis FitKids community-level demonstration project was to develop a locally relevant mobile-friendly web-based application to reach out to Memphis and Shelby County families to increase parental awareness about their child’s health habits, promote healthy lifestyles, improve children’s health, and in the long run, reduce the prevalence of overweight and obesity among children. The catchment area of this community-level project was Greater Memphis which includes all of Shelby County. The project aimed to develop and implement Memphis FitKids in the local community using the SM framework, track the traffic on the FitKids website, and evaluate the reach of FitKids through community events and Facebook. This paper describes the process of FitKids development, implementation, and the evaluation of the project.

## Methods

### A social marketing framework

FitKids utilized the SM framework to address the public health challenge of inactivity and unhealthy dietary behavior among children [47]. The SM framework includes the concept of a marketing mix consisting of product, price, place, and promotion [47]. The SM framework has been effectively used in numerous behavioral change interventions [31]. In public health, the ‘product’ denotes a health program or policy, a service, or behavior. In the case of FitKids the desired product was a healthy lifestyle among children (Table 1). The ‘price’ reflects the effort or cost to obtain the product. With regard to FitKids, this means relinquishment of some unhealthy habits; for example, reducing screen time or other sedentary behavior, or giving up some favored unhealthy foods. In the context of SM, the ‘place’ varies depending on the product; the place can be the location where people engage in new behaviors, receive services, or the distribution channels [47]. FitKids uses a mobile-friendly website, which uses a responsive web design to enable the tool to be used in smaller displays and touch screen devices, to distribute information and provides tools to facilitate healthy living. The last item of the marketing mix, ‘promotion’, typically refers to a combination of activities that support behavior change. FitKids used print (and printable) materials, incentives, community-based activities, and Facebook interaction to facilitate recognition of health issues with children and healthy lifestyles.

**Table 1:** FitKids social marketing strategy

Marketing Element	Definition	FitKids Application	Examples for FitKids
Product	The desired behavior	A healthy lifestyle	Happy children with healthy lifestyles (eating well, physically active, sufficient sleep, moderate amount of screen time) and low risks for developing metabolic diseases
Price	Monetary or nonmonetary cost for obtaining the benefits of the product	Relinquish convenient unhealthy habits to receive the benefits of the product	Reduce sugary beverages, increase the consumption of fruit and vegetables, reduce screen time and sedentary behavior, invest effort and time to become more physically active and do activities with the family
Place	The location or distribution channel where the product is offered or the service can be accessed	Internet	The mobile-friendly FitKids website http://memphisfitkids.org/ can be accessed with a variety of devices (phone, tablet, home computer, iPad) and at public access points (FitKids kiosks) in the community
Promotion	Strategies and activities designed to increase the awareness about the product or service and to promote the benefits of the product	Internet, print media, community events	Whiteboard animation, print materials, promotional items, banners, presentations at community events, and Facebook

#### Product

According to SM, the product is the desired behavioral change. FitKids’ product was a vision for healthy lifestyles for children and adolescents. The FitCheck tool increased parental awareness of health behaviors for both the child and the entire family including physical activity, diet, sleep, screen time, and family activities, based on established guidelines (Additional file 1). The recommendations for children and youth included 60 min or more of physical activity each day, and no more than 2 h of screen time per day [9, 48, 49]. Sleep and diet guidelines were based on the recommendations of the National Center for Chronic Disease and Prevention and Health Promotion and the 2015–2020 Guidelines of the U.S. Department of Health and Human Services and U.S. Department of Agriculture. Depending on the age of the child/teen the recommendations for sleep varied from 9 to 12 h, and for fruit and vegetable intake, they varied from 1 to 2 cups for fruit and 1–3 cups for vegetables [43, 45, 50–52]. The brief and clear instructions, and the visual cues in the personalized report (Additional file 2) generated by the FitCheck tool facilitated communication between parents and children on how to achieve the desired health behavior. The FitKids application encourages parents and their children to develop and maintain healthier routines by facilitating repeat assessments. To motivate parents to return to the website, they may establish a password-protected account that stores their FitCheck assessment data and allows them to track progress over time.

#### Price

Barriers for adopting a healthy lifestyle can include monetary cost such as a gym membership or nonmonetary cost such as giving up a favorite but unhealthy habit like watching TV for prolonged time. The FitKids’ GIS tool fostered a healthy lifestyle by providing coupons for healthy choices of local businesses and helping users to locate free or low-cost resources and activities in the community. This subtle “nudge” to parks, playgrounds, farmer’s markets and affordable, family oriented events could help FitKids users to overcome barriers for making healthier choices.

#### Place

FitKids was the place through which families could achieve the desired product, a ‘healthy lifestyle’. FitKids made it easier for families to receive meaningful information about their children’s health and supported lifestyle changes using various online platforms. FitKids was mobile-friendly and parents could access the Fitkids website (www.memphisfitkids.org) through their personal or public computer, phone, iPad, or at one of the five FitKids kiosks placed in the community (Additional file 3, Fig. 2).

**Fig. 2:**
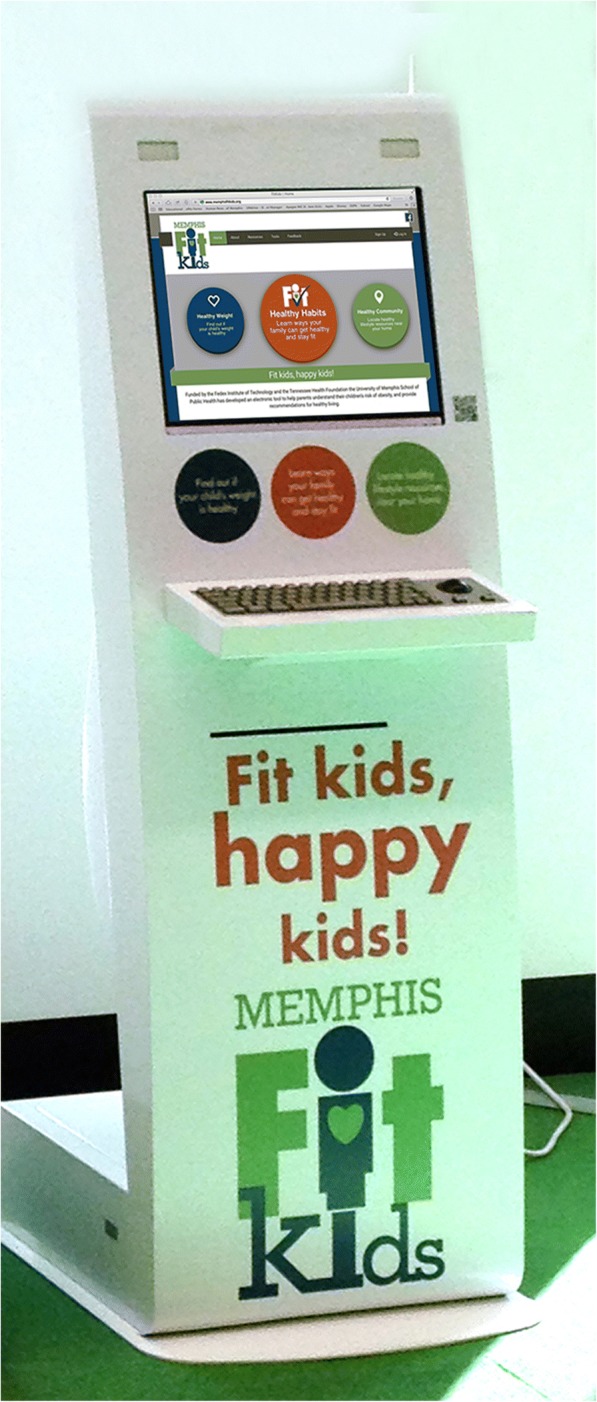
FitKids Kiosk

During the early stages of development, the FitKids system was extensively pilot-tested with parents, children, and several community organizations. The goal of the pilot project was to determine whether the system was easy for parents to understand and use, and whether it captured and generated information that parents considered to be meaningful and useful. Parents were shown a mockup website including behavioral questions and recommendations, a growth chart, and a GIS tool with a few community centers, fictitious activities and coupons; and they were asked what modifications to the system or additional information would be useful. Forty six parents were surveyed before and after they used the pilot system. All of the parents indicated that the screen had a “nice appearance.” Behavioral questions, behavioral recommendations, and the growth chart were easy to understand. Nearly all participants thought that the recommendations (98%) and the growth chart were useful (94%), and felt the length of the survey was appropriate (94%). Importantly, a majority of parents perceived that their knowledge of their child’s health risk was dramatically increased by taking the assessment, and indicated that they were interested in making positive behavioral changes, and sharing the information with their children’s healthcare providers. Based on the positive response to the one-year pilot testing, FitKids was further developed. The website upgrades included the design (e.g., logo, buttons), the content, and the functionality. The content was enhanced with an “About” page and a whiteboard animation with facts about FitKids, and a Resource page with information, video clips and web links related to healthy living. A Feedback button was created that allowed users to email FitKids. The design and functionality of the FitCheck report were improved by adding a disclaimer, graphics, colors and options for printing, emailing, and saving the report. The GIS tool, Healthy Community, added coupons, community centers, parks and playgrounds, and received additional features such as a search function and choices for filtering results. In addition, a multitude of Greater Memphis locations that facilitated physical activity and eating healthy were added to the map. On the back-end a database was created that allowed users to save their FitCheck reports, and the web development was refined to ensure that Fitkids ran on all common browsers and was mobile-friendly. The functionality to create a password-protected account was added which allowed users to store their FitCheck assessment and to track progress over time. The upgraded FitKids application was successfully implemented as a demonstration project in Memphis/Shelby County.

Several community organizations agreed to partner with the FitKids project. A Community Advisory Board (CAB), consisting of representatives from each of the community partners, advertising and marketing professionals, and academic partners from the University of Memphis, including the School of Public Health and the Center for Translational Informatics, was established. The CAB provided valuable input for the tool’s continuous modifications, development of a slogan, an attractive logo, and marketing messages, and facilitated its implementation in the community. With input from the CAB, locations for the kiosks were identified. Initially, some of the partners served as hosts for the kiosks carrying the FitKids program. Between 2015 and 2016, more FitKids partners were identified who were interested in hosting the kiosks. Therefore, additional kiosks were added and some kiosks were rotated among different partners to increase access points for the local parents.

#### Promotion

A variety of strategies were used to make the target audience - parents with children/youth between 2 to 20 years of age - aware about the FitKids tool and to promote the benefits of the product, a healthy lifestyle. A whiteboard animation, “FitKids, Happy Kids!” introducing all the FitKids features was implemented on the homepage of the website. The animation also ran on the kiosks to attract parents to engage with FitKids, and the animation was made available on the University of Memphis’ YouTube channel: https://www.youtube.com/watch?v=508-8v6TUas.

FitKids’ GIS tool provided coupons from community partners and local businesses to attract families to locations where they can be physically active or find healthy food sources (Additional file 4). Banners and print materials were displayed at community partner sites. Some of these items were tailored to specific locations (e.g., fliers in bookmark shape for a library, banners that point to a kiosk location). In addition, print materials were displayed in pediatric and family physician practices and were distributed at community events.

FitKids was routinely presented at numerous small and large community events such as farmers’ markets, health fairs, community walks and races, family days at museums, parks, schools, and faith-based institutions. Scales and tape measures were available, and project staff assisted parents in measuring their children’s height and weight. iPads were used to demonstrate the FitKids program. Having the iPads allowed interested parents to be guided through the FitKids application, and to answer any questions. In addition, parents and children received promotional items customized with the FitKids logo such as, pens, pencils, tattoos, bracelets, balls, and t-shirts. Apart from the tattoos, all items had the link to the FitKids website.

FitKids was also present on the social media platform, Facebook, to increase the reach of the target audience and to promote healthy choices for families (https://www.facebook.com/pages/Memphis-FitKids/290911627764809?_rdr). Regular posts emphasized the benefits of a healthy lifestyle, suggested fun activities for families in the community, and indicated where FitKids would be presented (example, see Additional file 5). The Facebook posts were prepared by graduate students of the School of Health Studies and the School of Public Health at the University of Memphis. Overall, the posts covered information about healthy nutrition, physical activity, family oriented community events, and places to look for FitKids kiosks and presentations. Facebook offered FitKids users the opportunity to engage with FitKids content, like or dislike posts, post comments, and send messages.

### Evaluation

A logic model was developed which guided the activities and progress of the FitKids project (Additional file 6). To track the traffic on the FitKids website and at the community access points where kiosks were located, Google Analytics was implemented. With Google Analytics the number of sessions and users were counted. Implemented filters allowed to focus on data of Tennessee users and to exclude bounced sessions. Bounced sessions are those where a request is made to a website, but no interaction with the website occurs. Google Analytics used the IP-address to identify users. If users accessed the FitKids website from different devices (e.g., iPad, phone, PC), Google Analytics was not able to identify them as returning users. A random pop-up survey invited FitKids website users to provide instant feedback. Social marketing efforts at various community events were logged and the distributed promotional items were counted to assess our reach in the community. Project staff at these events summarized in writing their observations and any feedback they received from the community members. Written comments were reviewed regularly by investigators and used to improve the FitKids website and future event participation. Facebook Insights is an instrument for tracking user interaction on Facebook pages and was used to evaluate the reach of Facebook posts.

## Results

**Fig. 3:**
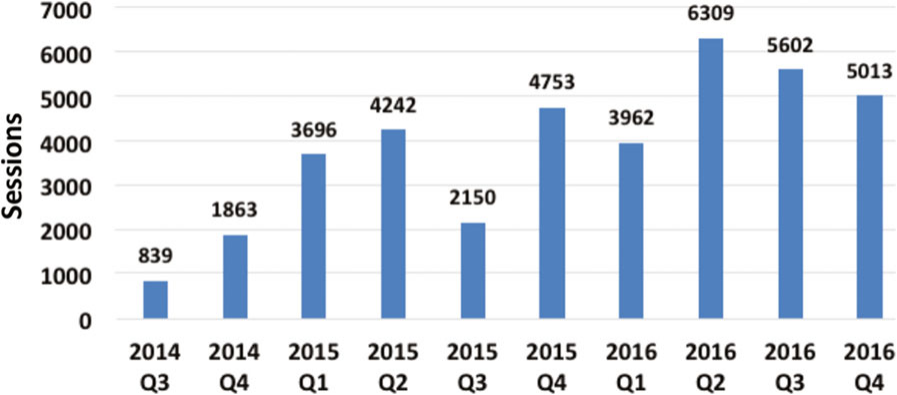
FitKids quarterly website sessions in tennessee 7/1/2014–12/31/2016

**Fig. 4:**
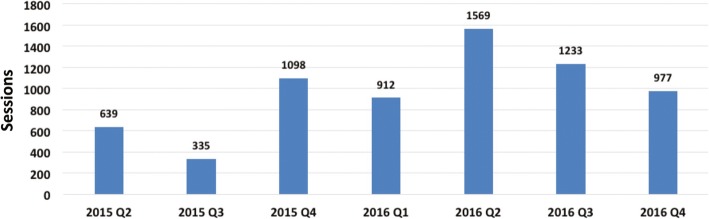
FitKids Kiosk sessions at locations in the greater Memphis’ community 4/1/2015–12/31/2016

Despite some seasonal variation, overall traffic on the FitKids website increased steadily. Based on Google Analytics estimates, between July 2014 and December 2016, 33,505 users in Tennessee completed 38,429 sessions (Fig. 3). Among these, 6763 sessions were completed at kiosks in Memphis (Fig. 4). Fig. 3 indicates an increase over time and some seasonal effects. Typically, the website traffic was lower in quarter 3 than in quarter 2 due to a decline around August. Data for the web traffic of the FitKids kiosks is available for the time period April 2015 to December 2016. The web traffic at these public access points also indicated less usage in quarter 3 than in quarter 2 (Fig. 4).

**Fig. 5:**
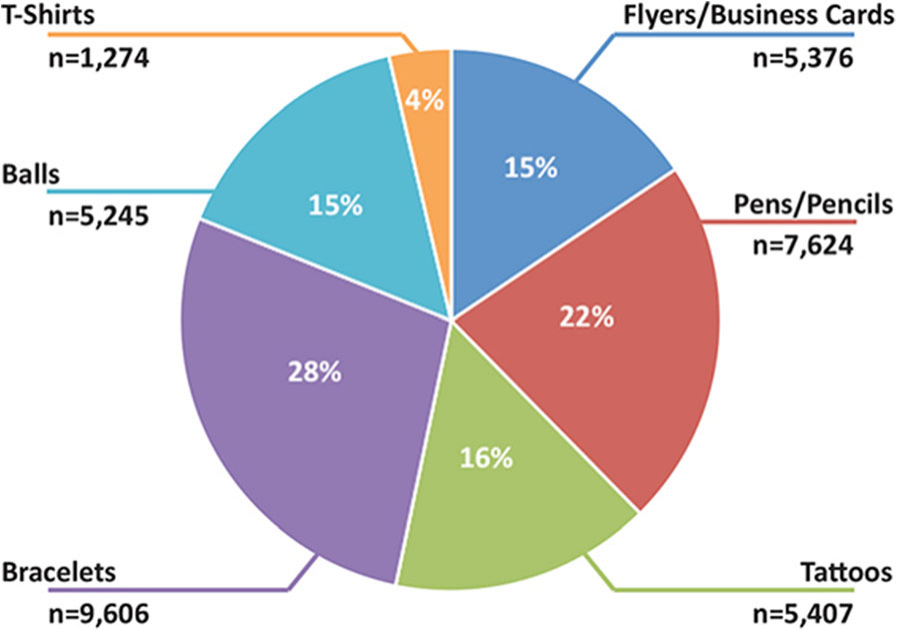
Distribution of promotional Items between 8/1/2014–12/31/2016

Between August 2014 and December 2016, FitKids was presented at 112 community events (e.g. farmer’s markets, big regional fairs, health fairs, family-oriented events at schools and faith-based institutions, and community runs/walks). Overall, 34,532 promotional items were distributed at these events (Fig. 5). The summaries provided by the project staff after each event included practical ideas (e.g., canopy weights needed to withstand wind), the acceptance of FitKids at specific events and FitKids user feedback. For example, at health fairs and family events in schools, parents were more likely to take the time to use the iPads, complete the FitCheck behavioral survey, and explore the FitKids resources than at big fairs. Families at fairs tended to pass by quickly and not stop for long, but would take promotional items before moving on to other exhibits or attractions. At school events teens were eager to receive a FitCheck report.

**Fig. 6:**
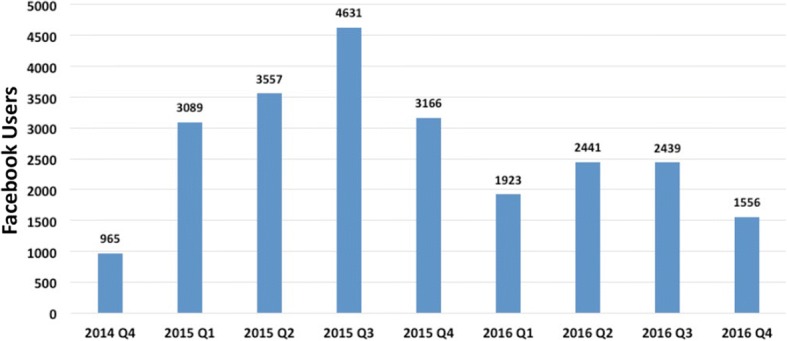
Quarterly reach of FitKids facebook posts

FitKids began using the social media site Facebook at the end of October 2014. First, FitKids started with two or three posts per week. Over time, the number of monthly posts increased, but reduced again in 2016 due to staffing issues. The number of posts per quarter varied from 43 to 222 with an average of 130 posts per quarter. Overall, 1168 posts were placed between October 2014 and December 2016, and these FitKids posts reached 23,767 unique Facebook users. Figure 6 shows the quarterly reach of all the FitKids Facebook posts across the three calendar years. On average each FitKids post reached 20 unique Facebook users.

## Discussion

The goal of this community-level demonstration project was to develop and implement FitKids - a mobile-friendly, web-based, child health application in the Greater Memphis area in Tennessee. The evaluation of FitKids’ implementation demonstrated that it had wide reach into the Memphis community, as indicated by 38,429 website sessions completed by 33,505 users, distribution of 34,532 promotional items to parents and children at 112 community events, and a Facebook presence that reached 23,767 unique users.

FitKids had several strengths, including the unique FitCheck tool generating tailored health behavior reports for children ages 2 to 20, and a custom-made GIS tool that directed parents to healthy lifestyle resources in their neighborhood and the surrounding community. To the best of our knowledge, FitKids is the first community-level application integrating GIS to serve families and children. The GIS tool with cues to farmers markets, activities, parks and playgrounds provided gentle nudges for families to harness neighborhood resources and showed that healthy choices can be inexpensive and easily accessible. Additional strength of FitKids is the multi-component approach that addressed several interlinked health behaviors such as nutrition, physical activity, sedentary behavior, and sleep patterns. Addressing multiple behavioral targets is considered essential to maximize the impact of health promotion interventions [53–56].

Another advantage of Fitkids is that it addressed a wide age range - two to twenty years, because little research is available targeting parents to improve health behaviors of their preschoolers [57]. Typically, parents have more influence on behavioral choices of preschoolers than later in childhood.

With regard to changing physical activity behavior at the community level, web-based and mobile-phone-based interventions appear to have positive effects [58]. The mobile-friendly web-based approach of Fitkids was consistent with user preferences as well with the widespread use of mobile phones in the United States [59–61]. The use of the internet was mentioned as the preferred channel for retrieving information about healthy food choices in a study with low-income mothers [59]. FitKids web traffic increased steadily and the Facebook page was well utilized.

The use of the social media channel Facebook to disseminate FitKids seemed appropriate as indicated by the wide reach of Facebook posts. Our finding is concordant with other studies that had used Facebook successfully to address health behaviors [62–65].

Feedback from FitKids users and community partners suggested that FitKids is widely accepted in the target population. Comparing the number of website sessions with the number of users suggested that many users did not return to the website. This was not surprising, given that the website was publically available, and it was expected that many users would not engage with it extensively (e.g., due to lack of interest or time). It is also possible that users were returning but we could not capture this because they used multiple IP addresses (e.g., accessed the website from their home computer, work computer, and/or smartphone).

Several issues may have reduced engagement with the website. First, FitKids had no features such as monthly newsletters or regular text messages to stay in contact continuously with users which seems to be important to reduce the fading of interest over time [25]. Second, FitKids had no features such as games to engage the children. Third, the implementation phase of FitKids overlapped with the development of the website due to the iterative process of website improvements which led to occasional instabilities of the website which may have challenged some users. Fourth, financial constraints did not allow a large scale multimedia campaign.

The effectiveness of social marketing campaigns has been challenged with the main argument that providing information alone does not necessarily lead to behavioral changes [66]. The intention of FitKids was not to replace other strategies such as clinical interventions instead it has been considered as complementing individual health behavior strategies. Social marketing campaigns have shown to influence behavior as one part of the strategy. The large well-financed VERB campaign succeeded in increasing children’s physical activity level and showed that parental awareness was linked to enhanced attitudes and beliefs about physical activity, the willingness to support their children’s physical activity, and to be physically active with them [42]. Common health behavior theories and models such as the Health Belief Model, the Transtheoretical Model or the Social Cognitive Theory consider people’s knowledge and awareness of health risks as initial requirements for aspired behavioral changes [67–69]. Results of the FitKids pilot project indicated that the FitCheck increased parents’ knowledge of their child’s health risk and that they were interested to make positive behavioral changes. This suggests that FitKids was successful in increasing users’ awareness about how families’ activity and diet habits can influence children’s health. Furthermore, the sensitive “nudges” of Fitkids’ FitCheck tool helped to reduce barriers to beneficial behaviors and showed how small changes can lead to healthier lifestyles and improve children’s health. If communitywide approaches like FitKids increase knowledge and awareness of health risks additional interventions paralleling or following these approaches may be more effective [70].

### Lessons learned

Mobile-friendly web-based applications like Memphis FitKids could be cost-effective and have promise for changing health behavior with large marketing campaigns using billboards, advertisements in local newspapers and magazines, and commercials in local broadcast media [38, 40, 71]. However, financial constraints of this demonstration project did not allow us to conduct a large-scale media campaign and to place more kiosks in the community. Mostly, free and low-cost marketing opportunities were pursued, including advertising through our community partners, and using social media.

FitKids was developed and implemented by a team of experts in public health, health promotion, marketing, graphic design, web design, and geographic information systems. Project staff were faculty, staff, and students of the University of Memphis. This setting had pros and cons. Utilizing university employees and students was very cost-effective compared to engaging private contractors. Also, engaging students and faculty nourished the project with fresh and creative ideas and allowed students to obtain valuable practical experience that enhanced their chances in the job market. On the other hand, working with students entailed staffing fluctuation and required regular hiring and training to replace students who graduated and moved on to steadier job opportunities. Likewise, faculty juggled multiple commitments, which sometimes slowed progress.

Talking with parents at community events made it clear that privacy was a very important issue. Parents were hesitant to use an electronic tool that may compromise their own or their child’s privacy. They wanted to be in charge and be able to decide who received the FitCheck reports. Parents were pleased to learn that FitKids gave them the option to use the tool either anonymously or by registering on the website.

### Future directions

FitKids, has been tested, refined, and the website traffic, the reach at community events and via Facebook indicated a successful implementation with the intention to improve children’s health. The FitCheck recommendations were based on best practice approaches and served the Greater Memphis community to educate parents about child obesity prevention. An advantage of FitKids is the scalability of this mobile-friendly web-based application; anyone worldwide can access the free website and generate tailored health behavior reports with the FitCheck tool of the application. The mobile-friendly version of Fitkids facilitated the reach of a large segment of the U. S. population due to the widespread use of cellphones [60]. This provides a foundation for any number of potential future uses of FitKids on a population or individual level. FitKids or features of Fitkids could be integrated in large health campaigns or healthcare providers could enhance existing health coaching programs with FitKids for families. FitKids could support health promotion efforts of non-profit as well as of for-profit health and fitness centers. In addition, the FitCheck tool and the FitKids resources could be utilized for pediatric/family practices, nutritionists, and health educators to support their clients’ efforts in achieving their health goals and track their progress. Using FitKids concurrently on the different levels (community, family and individual) may render beneficial effects [25]. In recent years, efforts have been made to emphasize the role of healthcare systems in the promotion of positive health behaviors, such as physical activity as a vital sign that should be monitored in clinical settings [72–74]. Web-based applications like FitKids can facilitate these initiatives.

Currently, the integrated GIS technology links parents to kid-friendly community resources in Memphis and Shelby County. Expansions to other geographic areas can be easily implemented. Additionally, FitKids can be adapted for other languages to serve the needs of non-English speaking groups. Recently, a Spanish version of the application has been completed for serving Memphis’ growing Latino population.

## Conclusions

The Memphis FitKids demonstration project is an exemplar of how a web-based health application can serve the health needs of a community. FitKids is potentially scalable and a viable strategy for promoting healthy behaviors. It holds the promise of increasing families’ awareness about health habits and the healthy lifestyle opportunities in their neighborhoods. Mobile health applications like Memphis FitKids could also serve health professionals in their efforts to support their patients and clients in achieving positive health behavior goals.

## Additional files

Additional file 1: FitCheck Healthy Habits Survey: Learn ways your family can get healthy and stay fit. (PDF 520 kb)

Additional file 2: FitKids - FitCheck report for parents. (JPG 471 kb)

Additional file 3: FitKids user with iPad. (JPG 394 kb)

Additional file 4: FitKids - Healthy Community Tool. (JPG 820 kb)

Additional file 5: FitKids Facebook post that reached more than 200 users. (JPG 679 kb)

Additional file 6: FitKids Logic Model. (JPG 889 kb)

## Abbreviations

GIS: Geographic Information Systems
SM: social marketing
