# Supplementary material for: Memphis FitKids: implementing a mobile-friendly web-based application to enhance parents’ participation in improving child health
Source: BMC Public Health. 2018 Aug 29;18:1068. doi: 10.1186/s12889-018-5968-6 (PMC6114180; doi:10.1186/s12889-018-5968-6)
Supplement: Supplementary file 1 — FitCheck Healthy Habits Survey: Learn ways your family can get healthy and stay fit. (PDF 520 kb) [file 12889_2018_5968_MOESM1_ESM.pdf]

FitCheck Questions	Response Options
Please enter the information below:	Enter child's name, Enter zip code (optional)
How old is your child today?	Ages 2-20
What is your child's gender?	Boy or Girl
What is your child's weight and height?	Enter weight(lbs) Enter height
Each week, how many days does your child eat breakfast?	0, 1, 2, 3, 4, 5, 6, 7
Each week, how many times does your child eat fast food?	0-1, 2-4, 5+
Each week, how many times does your family eat at the table together?	0, 1, 2, 3, 4, 5, 6, 7+
Each week, does your child drink more than one regular soda, sweet tea, fruit punch, or "sports drink"?	Yes or No
Most days, how many glasses of milk, cups of yogurt, or servings of cheese does your child eat or drink?	0, 1, 2, 3, 4, 5
What kind of milk does your child drink?	Skim, 1%, 2%, whole, other, none
Most days, does your child eat at least 5 servings of fruits and vegetables?	Yes or no
Most days, how much active play or sports does your child get, not counting time in school?	0-30min, 30-1hr, 1+hrs
Most days, how much time does your child spend watching TV, playing video games, text messaging, and using the computer?	0-1hr or 1+hrs
Most weeks, how many times does your family do something active together, such as hiking, walking, biking, or gardening?	0, 1, 2, 3, 4, 5, 6, 7
Does your child watch TV or use a computer in his/her bedroom?	Yes or No
Most nights, what time does your child go to bed?	7pm, 8pm, 9pm, 10pm, 11,pm, 12am, 1am, 2am, 3am
Most mornings, what time does your child wake up?	5am, 6am, 7am, 8am, 9am, 10am

FitCheck Recommendation Example

"Try to eat a sit-down breakfast together with [Child's name]. You're [his/her] role model. If you're rushed in the morning, offer yogurt or milk, a low-fat granola or breakfast bar, a hard-boiled egg or low-fat cheese, and even dinner 'leftovers' like chicken, fish, beans, and rice. If time runs out before leaving for school or work, give [Child's name] breakfast to eat on the way." And for increasing physical activity, FitKids suggests: "Kids who are active do better in school. Physical education (P.E.) and recess during school hours are a start, but kids need more activity in their free time. To get [Child's name] moving more, try

- Signing up [him/her] for recreational sports or a team,
- Suggesting 15 minute bursts of playing ball, jumping roping, bike riding, or dog walking. Minutes add up!
- Having [Child's name] join you on neighborhood walks or biking to the park.

Make being active fun! Fitness adds up to good health. Check out the 'Healthy Community' link at http://memphisfitkids.org/ for suggestions on where to be active in your neighborhood."
