# Supplementary figures and images for: Memphis FitKids: implementing a mobile-friendly web-based application to enhance parents’ participation in improving child health
Source: BMC Public Health. 2018 Aug 29;18:1068. doi: 10.1186/s12889-018-5968-6 (PMC6114180; doi:10.1186/s12889-018-5968-6)

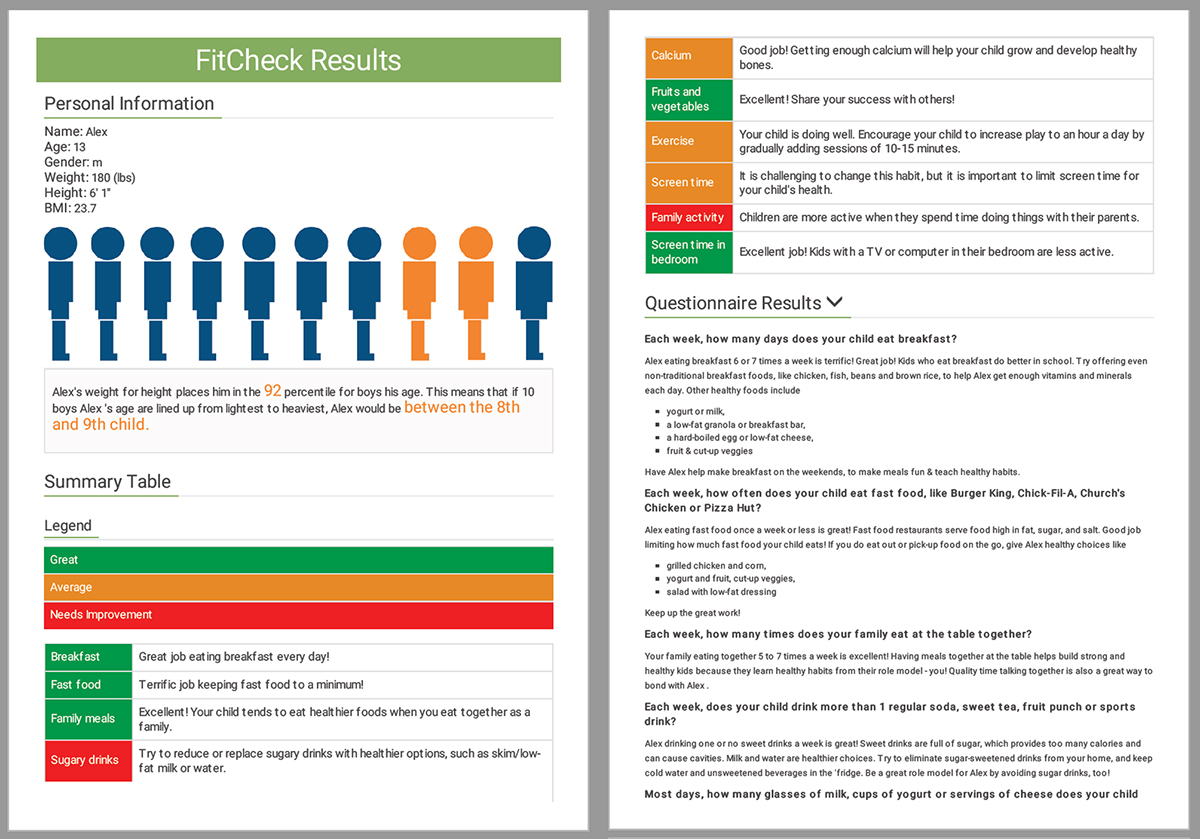

Supplement: Supplementary file 2 — FitKids - FitCheck report for parents. (JPG 471 kb) [file 12889_2018_5968_MOESM2_ESM.jpg]

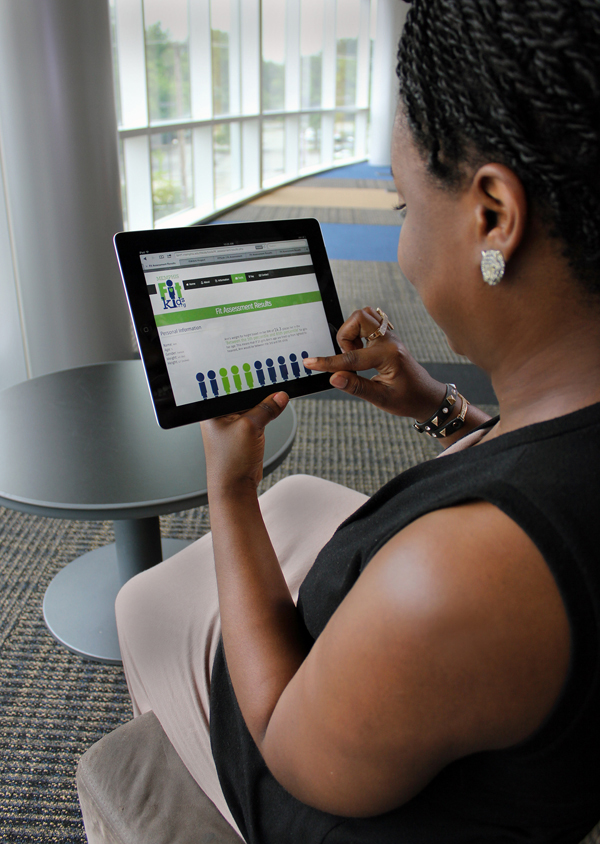

Supplement: Supplementary file 3 — FitKids user with iPad. (JPG 394 kb) [file 12889_2018_5968_MOESM3_ESM.jpg]

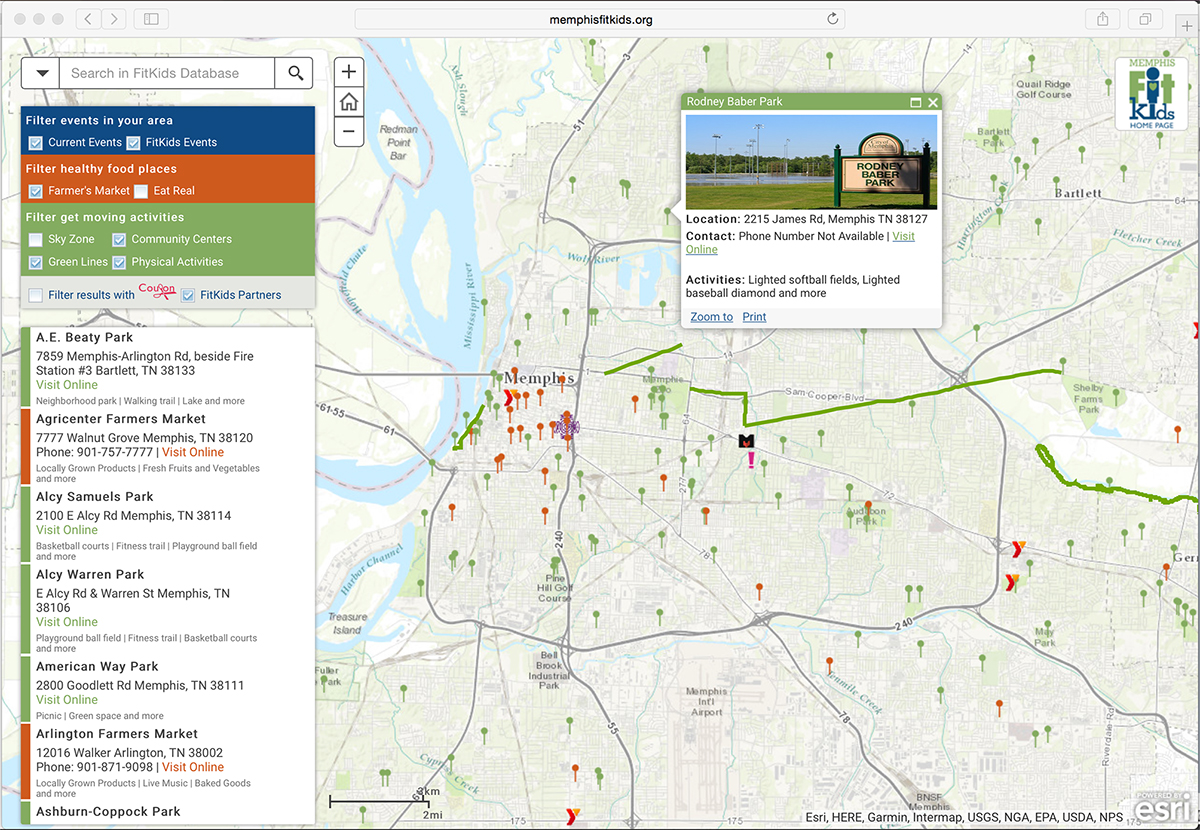

Supplement: Supplementary file 4 — FitKids - Healthy Community Tool. (JPG 820 kb) [file 12889_2018_5968_MOESM4_ESM.jpg]

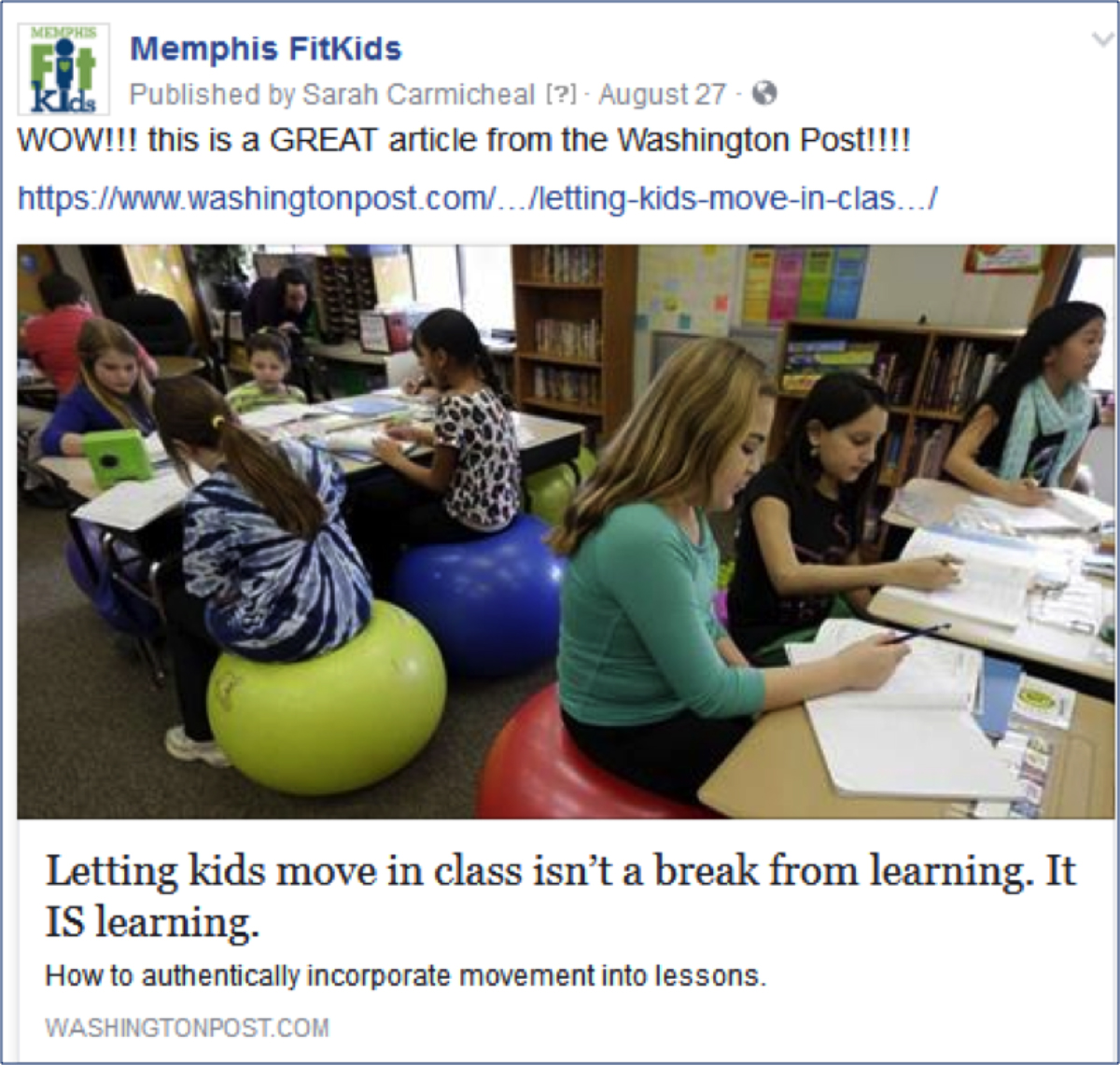

Supplement: Supplementary file 5 — FitKids Facebook post that reached more than 200 users. (JPG 679 kb) [file 12889_2018_5968_MOESM5_ESM.jpg]

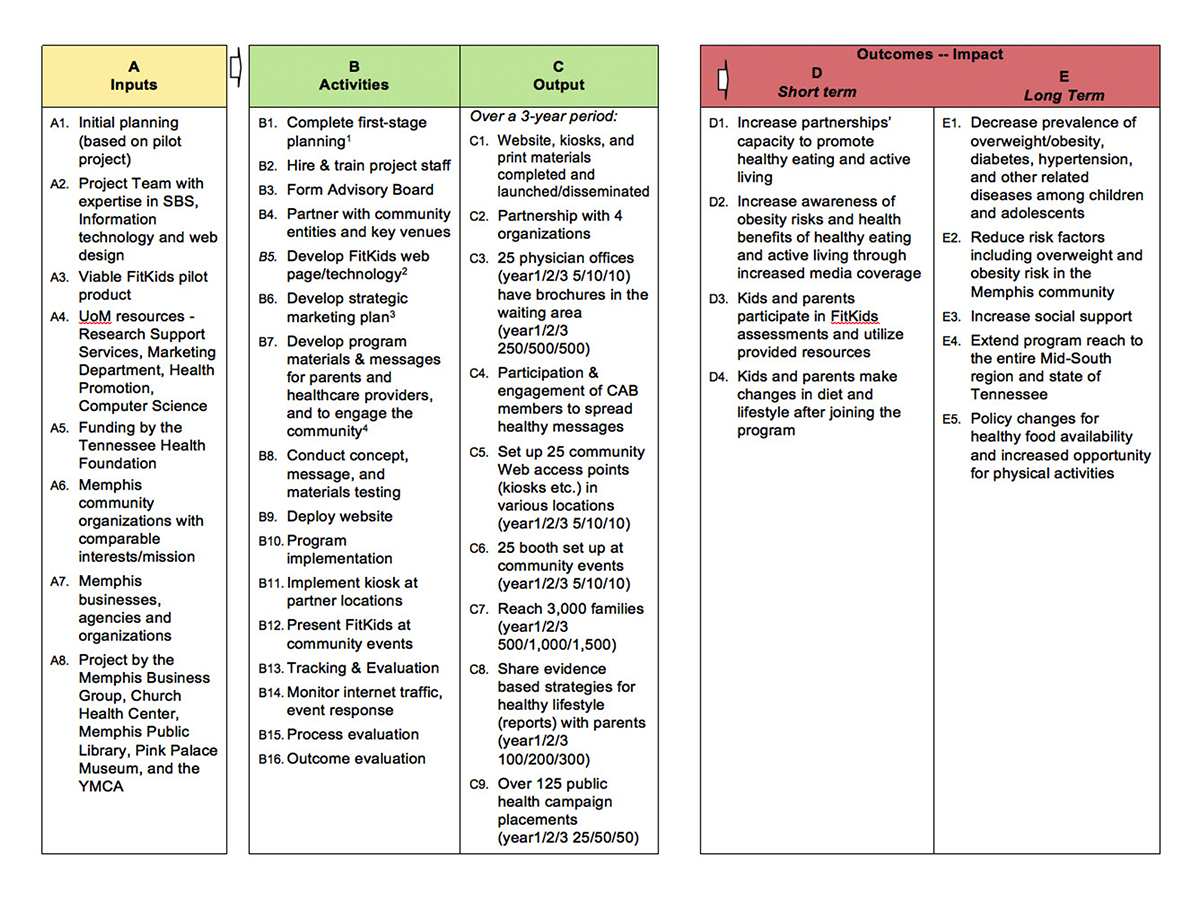

Supplement: Supplementary file 6 — FitKids Logic Model. (JPG 889 kb) [file 12889_2018_5968_MOESM6_ESM.jpg]
